# Outcomes of Multidrug-Resistant Tuberculosis Treatment with Early Initiation of Antiretroviral Therapy for HIV Co-Infected Patients in Lesotho

**DOI:** 10.1371/journal.pone.0046943

**Published:** 2012-10-24

**Authors:** Hind Satti, Megan M. McLaughlin, Bethany Hedt-Gauthier, Sidney S. Atwood, David B. Omotayo, Likhapha Ntlamelle, Kwonjune J. Seung

**Affiliations:** 1 Partners In Health, Maseru, Lesotho; 2 Department of Global Health and Social Medicine, Harvard Medical School, Boston, Massachusetts, United States of America; 3 Division of Global Health Equity, Brigham and Women’s Hospital, Boston, Massachusetts, United States of America; University of Cape Town, South Africa

## Abstract

**Background:**

Although the importance of concurrent treatment for multidrug-resistant tuberculosis (MDR-TB) and HIV co-infection has been increasingly recognized, there have been few studies reporting outcomes of MDR-TB and HIV co-treatment. We report final outcomes of comprehensive, integrated MDR-TB and HIV treatment in Lesotho and examine factors associated with death or treatment failure.

**Methods:**

We reviewed clinical charts of all adult patients who initiated MDR-TB treatment in Lesotho between January 2008 and September 2009. We calculated hazard ratios (HR) and used multivariable Cox proportional hazards regression to identify predictors of poor outcomes.

**Results:**

Of 134 confirmed MDR-TB patients, 83 (62%) were cured or completed treatment, 46 (34%) died, 3 (2%) transferred, 1 (1%) defaulted, and 1 (1%) failed treatment. Treatment outcomes did not differ significantly by HIV status. Among the 94 (70%) patients with HIV co-infection, 53% were already on antiretroviral therapy (ART) before MDR-TB treatment initiation, and 43% started ART a median of 16 days after the start of the MDR-TB regimen. Among HIV co-infected patients who died, those who had not started ART before MDR-TB treatment had a shorter median time to death (80 days vs. 138 days, p = 0.065). In multivariable analysis, predictors of increased hazard of failure or death were low and severely low body mass index (HR 2.75, 95% confidence interval [CI] 1.27–5.93; HR 5.50, 95% CI 2.38–12.69), and a history of working in South Africa (HR 2.37, 95% CI 1.24–4.52).

**Conclusions:**

Favorable outcomes can be achieved in co-infected patients using a community-based treatment model when both MDR-TB and HIV disease are treated concurrently and treatment is initiated promptly.

## Introduction

The convergence of the drug-resistant tuberculosis (DR-TB) and HIV epidemics represents a growing threat to public health. People living with HIV are particularly susceptible to TB infection and disease [1]–[3] and are often exposed to DR-TB while seeking care at hospitals and outpatient clinics. There have been many well-documented outbreaks of multidrug-resistant (MDR) TB among HIV-positive patients in Europe and the US [4]–[8]. Historically, DR-TB has not been thought to be a significant problem in African countries, many of which have generalized HIV epidemics, but most of these countries do not have the laboratory capacity for drug resistance surveillance [9]. Drug resistance surveys available from southern Africa suggest that the proportion of MDR-TB among TB cases in the region has increased during the past 15 years [9]. In the shocking discovery of extensively drug-resistant (XDR) TB in KwaZulu-Natal, South Africa, these patients were found to be almost exclusively HIV-positive [10].

Very little is known about the optimal treatment of patients with MDR-TB and HIV co-infection since most studies of MDR-TB treatment outcomes have been conducted in low HIV prevalence countries. Compared to first-line TB therapy, treatment for MDR-TB is lengthier and more complex, with a higher pill burden and greater risk of adverse effects from drug toxicity. HIV co-infection further complicates MDR-TB treatment because of the overlapping toxicities of antiretrovirals and second-line TB drugs [11], lack of knowledge about drug-drug interactions [12], and multiple potential causes of clinical deterioration during treatment [13], [14]. Despite the lack of scientific evidence, experts generally recognize the importance of an integrated response to HIV and MDR-TB [9], [15]–[17]. Recently updated World Health Organization (WHO) guidelines recommend prompt initiation of antiretroviral therapy (ART) for all co-infected MDR-TB patients, irrespective of CD4 cell count [18].

HIV-positive MDR-TB patients have been reported to have higher rates of mortality, treatment failure, and default than HIV-negative patients [19]–[23], but many of these studies were conducted before ART was widely available. A small number studies reporting outcomes of concurrent ART and DR-TB treatment have shown that ART improves the prognosis for co-infected patients [23]–[25]. We have previously reported early outcomes of MDR-TB treatment in Lesotho, where the majority of patients are HIV-positive [26]. Here we report final outcomes of comprehensive, integrated MDR-TB and HIV treatment in Lesotho and examine factors associated with increased hazard of death or failure.

## Methods

### Ethics Statement

This study was approved by the Partners HealthCare Human Research Committee. In the approved protocol, the requirement for informed consent was waived, since this was a retrospective study of information previously collected in the course of routine clinical care.

### Setting and Treatment Program

Lesotho, a mountainous country surrounded by the Republic of South Africa, faces a dual epidemic of TB and HIV. The estimated TB prevalence is 402 cases per 100,000 population [27], and the adult HIV prevalence is 24% [28]. Since 2007, the Ministry of Health and Social Welfare, with support from the non-governmental organization Partners In Health, has provided free diagnosis and treatment for patients with MDR-TB.

Patients with suspected MDR-TB who did not have drug susceptibility testing (DST) results at the time of referral were classified by risk level according to their treatment history and clinical, bacteriological, and radiological criteria [26]. High-risk patients were initiated on a standardized empiric regimen of six antituberculous drugs–pyrazinamide, kanamycin, levofloxacin, prothionamide (or ethionamide), cycloserine, and para-aminosalicylic acid–while awaiting culture and DST results. With few exceptions, patients began MDR-TB treatment within one week of their initial evaluation by clinicians in the national MDR-TB program.

The Lesotho National Reference TB Laboratory performed culture and DST for isoniazid, rifampicin, ethambutol, and streptomycin using a BACTEC MGIT 960 system (Becton-Dickson, Sparks, Maryland, USA). Specimens for smears and cultures were routinely collected at the initial evaluation, including sputum samples, pleural fluid specimens, and lymph node aspirates. Sputum induction with nebulized saline was used for patients who were unable to produce sputum at the initial evaluation.

All patients with unknown HIV status at the start of MDR-TB treatment were offered HIV counseling and testing. Co-infected patients who were not yet on ART began ART as soon as they are tolerating the MDR-TB regimen, regardless of CD4 cell count. The standard regimen consisted of two nucleoside reverse transcriptase inhibitors (lamivudine and zidovudine) and one non-nucleotide reverse transcriptase inhibitor (efavirenz).

The program integrated MDR-TB and HIV care at the community, clinic, and hospital levels [29]. Patients who were critically ill at the start of treatment or experienced severe adverse effects were hospitalized at a specialized inpatient facility in Maseru, where one clinical team managed HIV and MDR-TB for co-infected patients. When ready for discharge, a team of community nurses was responsible for assessing the home situation, educating the family, and arranging for a trained community health worker (CHW) to provide twice-daily DOT in the patient’s home for both antiretrovirals and second-line TB drugs.

The CHW also accompanied the patient to the clinic monthly for management of both HIV and MDR-TB at the same visit. During the monthly visit, patients underwent routine laboratory monitoring, including sputum smears and cultures, creatinine, electrolytes, and liver enzymes. CHWs received regular training on HIV and MDR-TB and close supervision, including surprise spot visits by the community nurses. The nurses rotated being on call to receive reports from the CHWs about any severe side effects or clinical deterioration experienced by the patients. CHWs were reimbursed for all costs incurred and compensated with performance-based payment that was dependent on their monthly evaluation.

The Lesotho MDR-TB program emphasized a holistic approach to patient care that included social, psychological, and economic support. Patients were not charged user fees in accordance with National TB Program guidelines. All patients received a food package and reimbursement for travel expenses related to treatment. CHWs and community nurses were trained in psychological support, and clinicians made every effort to provide humane and decent care without stigmatization.

### Patient Selection

In September 2011, we retrospectively reviewed the clinical charts of patients in the Lesotho national MDR-TB program who initiated second-line TB treatment between January 1, 2008 and September 29, 2009. Patients were included in the analysis if they had DST-confirmed MDR-TB (resistance to both isoniazid and rifampicin) and were 15 years of age or older. Patients were referred from all ten districts in Lesotho. Approximately one-third of patients were from Maseru district, the district surrounding the capital, and the remaining patients were distributed among the other nine districts.

### Statistical Analysis

We compared baseline characteristics between HIV-positive and HIV-negative patients using the Fisher’s exact test for dichotomous and categorical variables and the Wilcoxon rank-sum test for continuous variables. Standard definitions for retrospective MDR-TB analyses were used to determine final treatment outcomes [30]. Smear and culture results were considered baseline if they were from sputum samples taken within one month before or one week after initiation of MDR-TB treatment.

Cox proportional hazards modeling was used to assess potential risk factors for failure or death. We evaluated the proportional hazards assumption using the Kolmogorov-type supremum test. Patients who transferred out, defaulted, or completed treatment were censored at the date of their last visit. We imputed missing data using multiple imputation methods relying on all other variables. We used bivariable and multivariable analysis with each covariate, HIV status, and the interaction terms to identify confounding and effect modification. All variables examined in the bivariable analysis were considered as candidates for the multivariable model; interaction terms between baseline variables and HIV status that were significant at the p<0.1 level were also considered as candidates. We used a backward elimination process to build the final multivariable model. HIV status was retained in the final multivariable model, as it was the primary variable of interest. Other variables were retained in the final model if they were significant at the p<0.1 level in the multivariable model or if they changed the effect estimate for HIV status by more than 15%. SAS, version 9.2 (SAS Institute, Cary, North Carolina) was used for all analyses.

## Results

Between January 1, 2008 and September 29, 2009, 259 adult patients were initiated on treatment for confirmed or suspected MDR-TB. Seventy-seven patients were excluded from further analysis because they did not have DST results, and 48 patients were excluded from further analysis because DST revealed that they had pan-susceptible (n = 28), mono-resistant (n = 15), or poly-resistant, non-MDR (n = 5) strains. The remaining 134 patients with DST-confirmed MDR-TB were included in the study.

At baseline, 57 patients (42.5%) had low body mass index (BMI) (<18.5 kg/m^2^), and 19 (15.8%) had severely low BMI (<16 kg/m^2^). Thirty-five patients (26.2%) had been treated three or more times for TB, and 18 (13.4%) reported a history of treatment with second-line TB drugs. Most patients had severe radiographic findings: 118 patients (91.5%) had bilateral disease and 96 (74.4%) had cavitary disease or fibrosis. Table 1 presents the baseline clinical and demographic characteristics for the patients in this cohort by HIV status.

**Table 1:** Baseline demographic and clinical characteristics of MDR-TB patients, by HIV status.^a^

Characteristic		HIV-negative	HIV-positive	p^c^
		(N = 40)^b^	(N = 94)^b^	
Age (years)		43 (27.5–58.5)	39.5 (33–46)	0.3031
BMI (kg/m^2^)^d^	Severely low (<16)	7 (20.0)	12 (14.1)	0.4118
	Low (16 to <18.5)	13 (37.1)	25 (29.4)	
	Normal (≥18.5)	15 (42.9)	48 (56.5)	
Hemoglobin (g/dL)		12.1 (10.9–13.4)	11.1 (9.7–12.7)	0.0860
Albumin (g/L)		32.0 (27.5–36.0)	31.0 (27.0–37.0)	0.8366
Resting respiratory rate (breaths/min)		24 (23–28)	26 (24–28)	0.8292
Number of previous TB treatment regimens	None	1 (2.5)	4 (4.3)	0.5198
	One	10 (25.0)	33 (35.1)	
	Two	19 (47.5)	32 (34.0)	
	Three or more	10 (25.0)	25 (26.6)	
Time since first TB diagnosis (months)		29.9 (8.8–82.1)	19.0 (8.2–50.8)	0.2911
Sputum smear	Positive	7 (17.5)	8 (8.5)	0.2220
	Negative	22 (55.0)	50 (53.2)	
	Not available	11 (27.5)	36 (38.3)	
Number of drugs to which isolate was resistant	2 or 3	14 (35.0)	42 (44.7)	0.3420
	≥4	26 (65.0)	52 (55.3)	
Previous exposure to second-line TB drugs	Yes	2 (5.0)	16 (17.0)	0.0943
	No	38 (95.0)	78 (83.0)	
Fibrotic or cavitary lesions on chest radiograph	Yes	31 (79.5)	65 (72.2)	0.5106
	No	8 (20.5)	25 (27.8)	
Bilateral disease on chest radiograph	Yes	37 (94.9)	81 (90.0)	0.5027
	No	2 (5.1)	9 (10.0)	
Extrapulmonary TB	Yes	2 (5.0)	5 (5.3)	1.0000
	No	38 (95.0)	89 (94.7)	
Hospitalized at treatment initiation	Yes	10 (25.0)	30 (31.9)	0.5369
	No	30 (75.0)	64 (68.1)	
Sex	Male	25 (62.5)	54 (57.5)	0.7017
	Female	15 (37.5)	40 (42.6)	
Worked in South Africa	Yes	22 (56.4)	45 (48.9)	0.4515
	No	17 (43.6)	47 (51.1)	
Worked in South Africa as a miner	Yes	14 (35.9)	28 (31.5)	0.6842
	No	25 (64.1)	61 (68.5)	

**Note:** BMI: body mass index.

a Table values are median (IQR) for continuous variables and n (column %) for categorical variables.

b Numbers may not sum to total due to missing data, and percentages may not sum to 100% due to rounding.

c P-value is for Wilcoxon rank sum test (continuous variables) or Fisher’s exact test (categorical variables).

d For patients under 20 years of age, severely low BMI was defined as < −3 standard deviations, low BMI was defined as < −2 standard deviations, according to World Health Organization BMI-for-age charts for 5–19 years, 2–5 years.

Ninety-four (70.2%) patients were co-infected with HIV. At the start of the MDR-TB regimen, 50 (53.2%) of these patients were already on ART and had been on ART a median of 197 days (interquartile range [IQR], 91–511). Forty patients started ART after MDR-TB treatment was initiated, a median of 16 days (IQR, 6.5–31) later. Only four out of 94 patients never began ART, three of whom died within three months of starting MDR-TB treatment.

Among HIV co-infected patients who were tested within six months before or after initiation of MDR-TB treatment, median CD4 cell count was 220 cells/mm^3^ (IQR, 75–323). Of the 44 patients who were not on ART at the time of MDR-TB initiation, 33 had CD4 cell testing within six months before or after MDR-TB initiation. Of these, 9 patients (27.3%) had CD4 cell counts ≥350 cells/mm^3^, 7 (21.2%) between 200 and 349 cells/mm^3^, 11 (33.3%) between 50 and 199 cells/mm^3^, and 6 (18.2%) <50 cells/mm^3^.

Patients received MDR-TB treatment for a median of 22.9 months (IQR, 21.6–24.0) (Table 2). Treatment regimens were largely uniform–100% patients received a parenteral agent, 100% fluoroquinolone, 99.3% prothionamide (or ethionamide), 98.5% cycloserine, 95.5% pyrazinamide, 96.3% para-aminosalicylic acid, 2.3% terizidone, and 1.5% ethambutol. Kanamycin was the most commonly prescribed parenteral agent: 68.7% received kanamycin, 22.4% capreomycin, and 9.0% amikacin. Ofloxacin was the most commonly prescribed fluoroquinolone: 47.0% received ofloxacin, 42.5% levofloxacin, and 10.5% moxifloxacin. Forty patients (29.9%) were hospitalized at MDR-TB treatment initiation for a median of 34 days (IQR, 22–70), and 77 patients (57.7%) were hospitalized at some point during MDR-TB treatment.

**Table 2:** Clinical course and outcomes of MDR-TB treatment, by HIV status.^a^

			HIV-negative		HIV-positive	p^c^
				(N = 40)^b^	(N = 94)^b^	
Final outcomes	Cured			17 (42.5)	54 (57.5)	0.3613
	Treatment completed			4 (10.0)	8 (8.5)	
	Died			17 (42.5)	29 (30.9)	
	Defaulted			1 (2.5)	0 (0.0)	
	Failed			0 (0.0)	1 (1.1)	
	Transferred			1 (2.5)	2 (2.1)	
Treatment duration (months)				22.4 (21.6–24.0)	22.9 (21.8–23.9)	0.8838
Time on parenteral agent (months)^d^				10.2 (9.4–11.3)	10.5 (0.3–11.4)	0.6370
Time to culture conversion (days) (N = 84)^e^				59 (49–89)	66 (38–98)	0.6398
Time to smear conversion (days) (N = 79)^e^				48 (23–63)	66.5 (31–97)	0.0571
Time to death (days)				136 (86–340)	110 (66–208)	0.4169

a Table values are median (IQR) for continuous variables and n (column %) for categorical variables.

b Numbers may not sum to total due to missing data, and percentages may not sum to 100% due to rounding.

c P-value is for Wilcoxon rank sum test (continuous variables) or Fisher’s exact test (categorical variables).

d Calculated among those who completed the intensive phase of treatment.

e Calculated among those who had positive results at baseline (within one month before or one week after initiation of MDR-TB treatment).

The treatment success rate was 61.9%: 71 patients (53.0%) were cured, 12 (9.0%) completed treatment, 46 (34.3%) died, 3 (2.2%) transferred, 1 (0.8%) defaulted, and 1 (0.8%) failed treatment. Treatment outcomes did not differ significantly by HIV status (Table 2). Among patients with positive baseline cultures who converted, median time to culture conversion was 64.5 days (IQR, 38.5–95.0). Of those who died, 19 (41.3%) died within three months of treatment initiation, and 30 (65.2%) died within six months.

For those who were HIV co-infected, there was no association between hazard of death or failure and either CD4 cell count <200 cells/mm^3^ (hazard ratio [HR] 0.62, 95% confidence interval [CI] 0.23–1.68) or ART initiation prior to the start of MDR-TB treatment (HR 0.65, 95% CI 0.31–1.35). Although patients who started ART prior to MDR-TB treatment had a similar mortality rate compared with HIV co-infected patients who had not started ART before MDR-TB treatment (28% vs 34%, p = 0.655), those who were not already on ART had a significantly shorter median time to death (80 days vs. 138 days, p = 0.065).

In the final multivariable model, low or severely low BMI (HR 2.75, 95% CI 1.27–5.93; HR 5.50, 95% CI 2.38–12.69, respectively), and a history of working in South Africa (2.37, 95% CI 1.24–4.52) were significantly associated with greater hazard of death or failure (Table 3). HIV status was not a significant predictor of time to poor outcome in any of the models examined.

**Table 3:** Bivariable and multivariable associations between study variables and time to a poor treatment outcome (death or failure).

Characteristic		Unadjusted HR	Adjusted HR
		(95% CI)	(95% CI)
HIV status	Positive	0.66 (0.36–1.20)	0.77 (0.41–1.45)
	Negative	1.00	1.00
BMI (kg/m^2^) a	Severely low (<16)	4.99 (2.27–10.95)**	5.50 (2.38–12.69)**
	Low (16 to 18.49)	2.73 (1.30–5.75)**	2.75 (1.27–5.93)**
	Normal (≥18.5)	1.00	1.00
History of working in South Africa	Yes	1.82 (0.98–3.38)**	2.37 (1.24–4.52)**
	No	1.00	1.00
Hemoglobin (g/dL)	<10	1.58 (0.83–3.02)	1.18 (0.60–2.34)
	≥10	1.00	1.00
Age (years)	<35	1.00	–
	≥35	1.48 (0.75–2.92)	–
Sex	Male		–
	Female	1.00	–
Albumin (g/L)	<34	1.91 (0.84–4.36)	–
	≥34	1.00	–
Resting respiratory rate (breaths/min)	<30	1.00	–
	≥30	1.14 (0.57–2.26)	–
At least two previous TB treatments	Yes	1.50 (0.79–2.85)	–
	No	1.00	–
Previous exposure to second-line TB drugs	Yes	0.72 (0.28–1.83)	–
	No	1.00	–
Fibrotic or cavitary lesions on chest radiograph	Yes	1.20 (0.58–2.48)	–
	No	1.00	–
Bilateral disease on chest radiograph	Yes	2.30 (0.53–9.91)	–
	No	1.00	–
Extrapulmonary TB	Yes	1.61 (0.55–4.69)	–
	No	1.00	–

**Note:** HR: hazard ratio; CI: confidence interval; BMI: body mass index.

a For patients under 20 years of age, severely low BMI was defined as < −3 standard deviations, low BMI was defined as < −2 standard deviations, according to World Health Organization BMI-for-age charts for 5–19 years, 2–5 years.

** Significant at the p<0.1 level.

## Discussion

Despite the poor baseline clinical status of patients in this cohort, including a high rate of HIV co-infection, low BMI, and extensive lung damage, the treatment success rate was comparable to what has been reported in settings of low HIV prevalence [31]–[33]. Our findings demonstrate that good MDR-TB outcomes in the context of HIV are achievable using a community-based model. The traditional model of MDR-TB treatment in this region of the world has been hospital-based care, at least during the intensive phase of treatment, with the rationale that this model improves adherence, limits community transmission, and allows close monitoring, particularly for HIV co-infected patients who present more challenges for clinical management [34]. We largely attribute the success of this community-based program in Lesotho to the integration of MDR-TB and HIV care, empiric second-line TB treatment, and early ART initiation.

In Lesotho, CHWs were trained to administer both antiretrovirals and second-line TB drugs. A team of community nurses also provided strong communication between the CHWs and the clinic and hospital staff. CHWs were trained to recognize signs and symptoms of adverse effects requiring urgent referral to the clinic. Finally, patients were provided with food packages, transportation reimbursement, and other types of psychosocial support. These critical elements of community-based care for MDR-TB helped patients to transition successfully between the hospital and the community and finish the long treatment required for MDR-TB in their homes [29].

As a result, there was a very low rate of default (<1%) in this study population, in comparison to default rates exceeding 20% in programs in South Africa that have used inpatient care during the intensive phase and outpatient care without DOT during the continuation phase [19]–[21]. In other studies of MDR-TB treatment outcomes, high default rates may mask the true death or failure rate, since patients often default if they fail to improve clinically. Surprisingly, there was no significant difference in hazard of death or failure by HIV status in this cohort in Lesotho. Previous studies have reported high mortality rates among MDR-TB/HIV co-infected patients [19]–[23]. However, these studies either were undertaken before ART was available [20]–[22] or examined outcomes of a study population in which few patients were receiving ART [19].

In this study, more than 95% of co-infected patients received ART during MDR-TB treatment. Lesotho has made great strides in scaling up ART in recent years. Although there continue to be gaps in access, many more TB/HIV co-infected patients are diagnosed with HIV and appropriately started on ART compared to the early 2000’s. Of the 94 HIV-positive MDR-TB patients in this cohort, more than half had already been started on ART, usually during a previous course of TB treatment.

Recent WHO guidelines recommend that ART should be initiated 2–8 weeks after the start of MDR-TB treatment irrespective of CD4 cell count [18]. Dheda et al. showed that HIV-positive XDR-TB patients who never received ART had a higher mortality than those who were already on ART [25]. Palacios et al. found that ART confers a protective effect for HIV co-infected MDR-TB patients, but the median time from the start of MDR-TB treatment to ART initiation exceeded five months [23]. In this cohort in Lesotho, nearly all HIV-positive patients not yet on ART were initiated on ART within several weeks of starting MDR-TB treatment. Nevertheless, the time to death in HIV-positive patients who were not already on ART prior to MDR-TB treatment was significantly shorter compared to HIV-positive patients who were already on ART. This indicates that early initiation of ART is indeed important–patients who are severely immunosuppressed may take several months to show a benefit from ART.

Another reason for the improved outcomes among HIV-positive patients compared to HIV-negative patients may have been the empirical use of second-line TB drugs, which international guidelines recommend for patients with a high likelihood of MDR-TB [35]–[37]. If culture-based DST methods are used, as was the case for Lesotho during the study period, it may take months for DST results to become available. Studies from KwaZulu-Natal, South Africa have found that when DR-TB/HIV co-infected patients were treated with standard first-line TB therapy while awaiting DST results, many did not survive long enough to initiate appropriate treatment [19], [34]. While the increasing access to rapid molecular methods of DST may make empiric MDR-TB therapy less necessary in the future, the findings from these studies underscore the need for rapid diagnosis of DR-TB and early initiation of second-line TB drugs in settings with high HIV prevalence.

Finally, survival bias or referral bias may have affected the baseline characteristics and outcomes of HIV co-infected patients in this cohort. Unmeasured factors that affected the DR-TB/HIV co-infected patients’ ability to survive until referral and evaluation for MDR-TB might lessen the effect of HIV on MDR-TB treatment outcomes. Since many of the HIV co-infected patients were already in ART or pre-ART care at the time they were referred for evaluation of MDR-TB, they may have been identified and referred at an earlier stage in the progression of their TB disease compared to patients without HIV co-infection, although they did not differ significantly on measured signs of severe, protracted disease (low BMI, bilateral disease, fibrotic or cavitary disease, or elevated respiratory rate).

Our multivariable analysis revealed several other factors associated with increased risk of poor outcomes. First, similar to previous studies [20], [31], [38], we found a strong, independent association between low BMI at baseline and increased risk of death or failure. Because low BMI is a sign of severe TB or HIV disease, the strong association between low BMI and greater hazard of death or failure is not surprising and underscores the importance of early initiation of appropriate therapy. Second, a history of working in South Africa was also a significant, independent predictor of greater hazard of death or failure. A significant proportion of the male population in Lesotho–an estimated 50,000 men annually–work in South African mines, and the occupational hazards and social context of mining puts them at greater risk for acquiring TB [39], [40]. Additionally, living in South Africa may affect patients’ health care access, health care seeking, and treatment history prior to referral for suspected MDR-TB, which in turn may affect unmeasured baseline clinical characteristics that have implications for MDR-TB treatment outcomes.

This study used data collected during the course of routine clinical practice. Some patients were missing DST results, because of negative or contaminated baseline cultures resulting from issues at the national laboratory, and were excluded from the study. The Lesotho National Reference TB Laboratory did not have the capacity to perform DST for second-line antituberculous drugs, so the prevalence of XDR-TB among the patients in this cohort is unknown. Given the proximity to KwaZulu-Natal, a proportion of the deaths in this cohort may have been attributable to XDR-TB [10]. Another limitation is that some patient charts were missing additional baseline data, notably height (10%), albumin (22%) and hemoglobin (6%). We imputed this data, assuming the data are missing at random, which is plausible but untestable with the data. The general conclusions did not change when we restricted to a complete analysis.

The Lesotho MDR-TB treatment model is comprehensive. It includes strong links between community-based, clinic and inpatient care; DOT throughout treatment; empiric use of second-line TB drugs while awaiting DST results; and concurrent ART and MDR-TB treatment for more than 95% of co-infected patients. The treatment success rate for this program in a high HIV-prevalence setting is comparable to those reported from settings of low HIV prevalence. More research on the clinical management and outcomes of MDR-TB/HIV co-infected patients treated concurrently for MDR-TB and HIV disease is needed. Future studies should evaluate the effect of timing of ART after initiation of MDR-TB treatment on treatment outcomes, frequency and severity of adverse reactions, and incidence of immune reconstitution inflammatory syndrome. Nevertheless, the findings from Lesotho suggest that favorable outcomes can be achieved in co-infected patients when both MDR-TB and HIV disease are treated concurrently and treatment is initiated promptly.
